# Folate Receptor Alpha Autoantibodies in Vector-Borne Disease Populations

**DOI:** 10.3390/diseases14060202

**Published:** 2026-06-05

**Authors:** Lindsey Wells, Myriah Hinchey, Richard E. Frye, Amelia Morgan

**Affiliations:** Autism Discovery and Treatment Foundation, Phoenix, AZ 85050, USA; drhinchey@taovitality.com (M.H.); drfrye@autismdiscovery.org (R.E.F.); ameliamorg4@gmail.com (A.M.)

**Keywords:** folate receptor alpha autoantibodies, folate receptor alpha, leucovorin, pediatric acute-onset neuropsychiatric syndrome (PANS), vector-borne diseases

## Abstract

Background: Vector-borne diseases (VBDs) caused by *Borrelia* spp., *Bartonella* spp., and *Babesia* spp. are associated with neuropsychiatric morbidity. Cerebral folate deficiency (CFD), primarily caused by folate receptor-α autoantibodies (FRAAs) impairing folate blood–brain barrier transport, is a treatable contributor to neurodevelopmental disorders including pediatric acute-onset neuropsychiatric syndrome (PANS) and autism spectrum disorder. Despite overlapping clinical manifestations, FRAA prevalence in VBD populations has not been investigated. This study aimed to determine the prevalence of FRAA in patients with confirmed VBDs. Methods: This retrospective cohort study included 68 VBD-positive patients with and without PANS evaluated at a single clinical practice. VBD testing was performed by IGeneX Laboratories; FRAA analysis including binding, blocking, and soluble folate receptor (sFR) testing was performed by Religen Laboratories (Plymouth Meeting, PA). Statistical associations were assessed using Fisher’s exact test with Wilson 95% confidence intervals. Results: Of the 68 VBD-positive patients, 42 (61.8%; 95% CI: 49.9–72.4%) were also FRAA-positive. Soluble folate receptor (sFR) was detected in eight patients (11.8%; 95% CI: 6.1–21.5%), all of whom were binding FRAA-positive, with 87.5% carrying confirmed evidence of *Borrelia* species infection. Neuropsychiatric symptoms were highly prevalent across both groups but did not significantly differentiate FRAA-positive from FRAA-negative patients (all *p* > 0.05). Conclusions: This study demonstrates a high prevalence of FRAA in a pediatric VBD cohort. The sFR was strongly associated with *Borrelia* species infection, suggesting a potential mechanistic link between spirochetal infection and folate receptor autoimmunity. These findings support the consideration of FRAA testing in patients with VBDs and warrant further investigation in larger prospective cohorts.

## 1. Introduction

Cerebral folate deficiency (CFD) is defined by below-normal 5-methyltetrahydrofolate (5-MTHF) levels in the cerebrospinal fluid (CSF) despite normal 5-MTHF concentrations in the serum [1,2]. Under normal physiological conditions, folate receptor-α (FRα) facilitates receptor-mediated endocytosis of 5-MTHF across the blood–brain barrier. CFD is caused by impairment in this folate transport [3,4]. The main cause of this disruption is FRα autoantibodies (FRAAs).

Two types of FRAAs have been identified: blocking and binding FRAAs. The blocking FRAA binds to the folate-binding site of the FRα, preventing 5-MTHF from binding to the receptor. The binding FRAA binds other regions of FRα and interferes with receptor function [5,6,7]. This contributes to abnormalities in one-carbon metabolism, mitochondrial function, serotonin and dopamine synthesis, and neuroinflammation in the brain [8,9,10].

CFD has been documented in several neuropsychiatric disorders. CFD has been identified in 36% of patients with treatment-resistant depression (TRD). When TRD patients with documented CFD received leucovorin (folinic acid) treatment alongside their antidepressants, many experienced substantial symptomatic improvements [11]. Another study found that 9 out of 16 patients with TRD were positive for FRAAs [12]. CFD due to FRAAs has also been reported in 83.5% of patients with refractory schizophrenia, with leucovorin treatment stabilizing the disease process [13].

Vector-borne diseases (VBDs), including the *Borrelia burgdorferi sensu lato* complex, *Bartonella* spp., and *Babesia* spp., present with diverse symptoms that range from early acute localized infection to chronic late disseminated disease with multi-system involvement. For example, in Lyme disease, symptoms include constitutional features such as fatigue, malaise, and fever; musculoskeletal pain including migratory arthralgias, myalgias, and arthritis; neuropathic pain; cardiac conduction disturbances; dysautonomia; sensory hypersensitivities; and sleep disturbances [14,15].

VBDs are also associated with neuropsychiatric morbidity. In a national Danish cohort study, Lyme borreliosis was associated with significantly increased risk of any mental disorder, affective disorders, suicide attempts, and suicide mortality [16]. Another study reported elevated rates of depression, anxiety, and sleep disturbances in patients with post-treatment Lyme disease syndrome (PTLDS), a condition that impacts 10–20% of patients with confirmed Lyme disease treated with antibiotics [17]. Another study reported that 37% of Lyme disease patients scored in the lower third of a quality-of-life scale and showed cognitive impairments [17]. In PTLDS, neuroimaging reveals white-matter changes in the frontal lobe that contribute to cognitive difficulties [18]. Exposure to *Bartonella* spp. is disproportionately reported in individuals with schizophrenia and schizoaffective disorder, with molecular and serologic evidence suggesting a three-fold higher prevalence compared with controls [19]. Case-based evidence links *Bartonella* bacteremia to agitation, panic attacks, and treatment-resistant depression [20]. Critically, *Bartonella henselae* has been directly visualized in resected brain tissue using confocal laser scanning microscopy and was independently confirmed by PCR in a pediatric patient with a 12-year history of progressive neurological symptoms including hallucinations, seizures, paralysis, and cognitive dysfunction, providing histopathological evidence of cerebral *Bartonella* infection [21]. Neuropsychiatric manifestations are described in relapsing-fever borreliosis and babesiosis, including encephalopathy, mood symptoms, and altered cognition [22,23].

VBDs induce neuropsychiatric disorders by triggering an immune response. Multiple pathological mechanisms contribute to the development of neuropsychiatric symptoms. For example, following tick inoculation, *B. burgdorferi* (s.l.) engages Toll-like receptors and induces matrix metalloproteinases (including MMP-9), leading to disruption of endothelial tight-junction proteins, basement membrane degradation, and increased blood–brain barrier (BBB) permeability [15,24]. In Lyme neuroborreliosis, CSF demonstrates elevated interleukin-6 (IL-6), C-X-C motif chemokine ligand 13 (CXCL13), and intrathecal IgG synthesis consistent with robust B-cell recruitment and germinal center activity, alongside Th17-skewed immunity that amplifies inflammatory signaling and tissue injury [25,26]. Positron emission tomography (PET) neuroimaging confirms persistent microglial activation in post-infectious Lyme patients, supporting a chronic glial-primed neuroimmune phenotype [27]. Collectively, these mechanisms illustrate how *Borrelia* infection can produce sustained neuroimmune activation that contributes to neuropsychiatric symptomatology.

Similarly, *Bartonella henselae* exhibits tropism for endothelial cells, erythrocytes, and pericytes, promoting endothelial inflammation, vascular endothelial growth factor (VEGF)-mediated angiogenesis, microvascular instability, and neurovascular injury [28,29]. Comparable to *Borrelia*, *Bartonella* infection can induce IL-6–rich inflammatory signaling, mitochondrial stress, and Th17-polarizing cytokine pathways, contributing to persistent immune activation and vascular–neural interface dysfunction. Endothelial inflammation and vasculopathic lesions further support microvascular CNS stress as a driver of neuropsychiatric and autonomic instability. These overlapping processes reinforce the capacity of *Bartonella* infection to contribute to neuropsychiatric presentations through immune-mediated and vascular-neural mechanisms [28,30].

*Babesia microti*, an intraerythrocytic apicomplexan parasite, drives hemolysis, complement activation, endothelial injury, mitochondrial dysfunction, and the release of pro-inflammatory cytokines including IL-6 and tumor necrosis factor-alpha (TNF-α), amplifying systemic and central nervous system (CNS) immune activation [31,32,33]. Although biologically distinct from *Borrelia* and *Bartonella*, these immunologic and vascular effects support a mechanism by which babesiosis may contribute to neuropsychiatric symptoms [23].

Pediatric acute-onset neuropsychiatric syndrome (PANS), a post-infectious autoimmune disorder, is characterized by the abrupt onset of neuropsychiatric symptoms following infections such as *Streptococcus*, *Mycoplasma pneumoniae*, influenza, COVID-19, and other viral and bacterial triggers [34]. In PANS, FRAAs are detected in 63.8% of affected individuals [35]. One case report further demonstrated that leucovorin treatment was associated with improvements in anxiety, OCD, and executive functioning in an FRAA-positive PANS patient [35].

The overlap between neuropsychiatric profiles in VBDs and those observed in CFD-positive TRD, psychosis, and PANS suggest a shared immunopathologic mechanism mediated by CFD. Despite this biologically plausible link, no study has investigated FRAA prevalence in patients with VBDs. Identification of such an association could inform risk stratification and support targeted metabolic correction of CSF 5-MTHF concentrations using leucovorin, a low-risk therapeutic intervention with demonstrated efficacy in FRAA-associated disease.

The objective of this study was to determine the prevalence of FRAAs in patients with confirmed VBDs.

## 2. Methods and Materials

### 2.1. Subjects

This retrospective cohort study was conducted at Lindsey Wells ND, LLC, a single naturopathic medicine practice located in Wilton, Connecticut, from January 2022 to September 2024, and subsequently in Trumbull, Connecticut, from October 2024 to August 2025. Data were collected from patients evaluated between January 2022 and August 2025.

Patients were evaluated, tested, and diagnosed by the first author (LW). Laboratory values and symptomatology were abstracted from records into a deidentified database. This study was determined to be exempt under 45 CFR § 46.104(d)(4) by the WCG IRB (Puyallup, WA, USA). Symptoms consistent with anxiety, OCD, depression, ASD, attention-deficit/hyperactivity disorder (ADHD), PANS, and pediatric autoimmune neuropsychiatric disorders associated with streptococcal infections (PANDAS) were rated as present or not present.

### 2.2. Folate Receptor Alpha Autoantibody Assay

Approximately 2–4 mL of blood was drawn into a serum-separating tube and separated by centrifugation. A 2 mL aliquot of serum was transferred to a transport tube and shipped to Religen Laboratory (Plymouth Meeting, PA, USA) for analysis. The FRAA assay was performed by a Clinical Laboratory Improvement Amendments (CLIA)-certified pathway. The assay reported three results: serum-binding titer, serum-blocking titer, and the presence or absence of soluble folate-binding proteins.

The binding titer measures the binding of IgG antibodies to the FRα using an enzyme-linked immunosorbent assay (ELISA) as previously mentioned [7]. The blocking assay specific for binding IgG is used to measure binding FRAAs as previously mentioned [7]. The blocking assay uses an in vitro assay to measure the blood concertation of autoantibody that is specifically blocking the binding site for folate on the FRα. The amount of radiolabeled folate displaced from the FRα when the patient’s serum is added is measured in this assay [7].

### 2.3. IGENEX

To detect the specific VBD, infection was measured using the IGeneX Laboratory (Milpitas, CA, USA). The IGeneX ImmunoBlot (IB) assay is a qualitative serologic method employing recombinant antigens to detect pathogen-specific antibodies. This test was used to detect four vector-borne pathogens—*Borrelia burgdorferi*, relapsing-fever *Borrelia* (TBRF), *Babesia*, and *Bartonella*—using patient serum. For the Lyme IB laboratory test, recombinant proteins representing multiple *Borrelia* species and strains were immobilized at defined positions on a nitrocellulose membrane, which was sectioned into individual strips. Patient serum was incubated with the strips to allow for the binding of *B. burgdorferi*-specific antibodies, which were subsequently visualized. IgM results were classified as positive if two or more of the following bands were detected: 31, 39, 41, or 43 kDa. IgG results were considered positive if two or more bands (23, 31, 34, 41, or 93 kDa) were detected. For both the IgG and IgM IB results, a single band was classified as indeterminate, and the absence of bands was classified as negative.

The TBRF IB is a qualitative assay for detecting IgM/IgG antibodies from TBRF-associated *Borrelia* species (*B. miyamotoi*, *B. hermsii*, *B. turicatae*, and *B. coriaceae*) using recombinant antigens immobilized on nitrocellulose strips incubated with patient serum. The criteria for a positive IgG and IgM test result included the detection of 2 or more TBRF *Borrelia* species-specific antibodies. This same IB assay was also used to detect IgM/IgG antibodies from multiple *Bartonella* species, including *B. henselae*, *B. quintana*, *B. elizabethae*, and *B. vinsonii*. The criteria for a positive IgG and IgM test result included the detection of 2 or more *Bartonella* species-specific antibodies.

The *Babesia* IB assay was also used to test for IgG/IgM antibodies from the *Babesia* genus; this assay differentiates among several *Babesia* species known to infect humans, including *Babesia microti* and *Babesia duncani*. The criteria for a positive IgG and IgM test result included the detection of 2 or more *Babesia* species-specific antibodies. A negative result occurred when only one *Babesia*-specific antibody was detected or in the event of the complete absence of a *Babesia*-specific antibody.

The *Babesia* Fluorescent In Situ Hybridization (FISH) assay was used to test for the direct detection of *Babesia* ribosomal RNA in blood smears using fluorescein-labeled, species-inclusive nucleic acid probes [32,33]. The assay involves probe hybridization with *Babesia*-specific RNA on thin blood smears and visualization by fluorescence microscopy, with an LED attachment containing 492 nm excitation and 530 nm emission band-pass filters [32,33]. A positive result is defined by the formation of a fluorescing ring in at least two red blood cells (RBCs); a negative result is defined by the absence of fluorescence within the RBCs. The FISH assay was also used to detect *Bartonella* ribosomal RNA in patient blood smears. The same positive and negative criteria applied to patient RBCs for the detection of *Bartonella* ribosomal RNA. A positive result of the *Bartonella* FISH assay indicated the presence of bacteria from the genus *Bartonella*, including species such as *B. berkhoffii*, *B. henselae*, *B. elizabethae*, *B. quintana*, *B. vinsonii*, and others.

### 2.4. Statistical Analysis

All statistical analyses were performed using Python (version 3.x). Continuous variables are reported as mean ± standard deviation (SD) with range, and categorical variables are expressed as frequencies and percentages. This analysis was restricted to the 68 VBD-positive patients, as the primary objective was to determine the prevalence of FRAAs in a confirmed VBD population. FRAA prevalence and soluble folate receptor (sFR) detection rates are reported with 95% confidence intervals (CI) calculated using the Wilson score method. Differences in neuropsychiatric symptom prevalence between FRAA-positive (*n* = 42) and FRAA-negative (*n* = 26) patients were assessed using Fisher’s exact test, which was selected over the chi-square test given the small sample size in several subgroups. The association between PANS/PANDAS diagnosis and FRAA status was similarly assessed using Fisher’s exact test, with the odds ratio and 95% confidence interval calculated using the Woolf method. A *p*-value of <0.05 was considered statistically significant for all comparisons. No corrections for multiple comparisons were applied given the exploratory and hypothesis-generating nature of this study.

## 3. Results

### 3.1. Patient Characteristics

A total of 68 patients with VBDs were enrolled in this retrospective cohort study. The cohort consisted of 43 males (63.2%) and 25 females (36.8%), with a mean age of 12.8 years (SD ± 6.7; median 13 years; range 2–31 years). The majority were pediatric patients (*n* = 54, 79.4%), with 14 young adults (20.6%) (Table 1).

### 3.2. Prevalence of FRAAs

Of the 68 VBD-positive patients tested, 42 (61.8%; 95% CI: 49.9–72.4%) had detectable FRAAs.

### 3.3. Soluble Folate Receptor (sFR) Analysis

**Figure 1:**
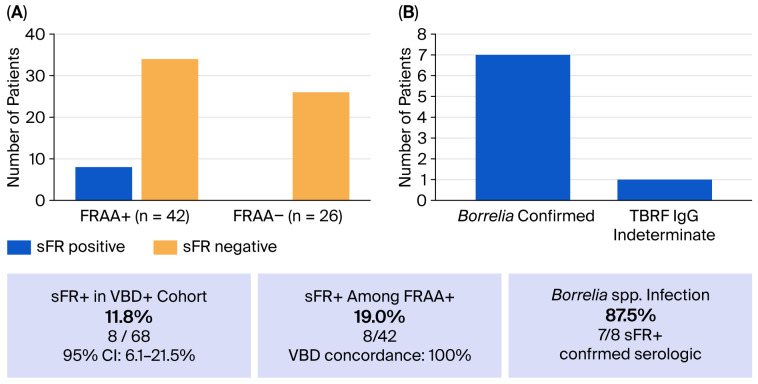
Soluble folate receptor (sFR) status and associated *Borrelia* species infection patterns in a VBD-positive cohort evaluated at Lindsey Wells ND, LLC, Wilton and Trumbull, Connecticut, January 2022–August 2025 (*N* = 68). (**A**) sFR status stratified by folate receptor alpha autoantibody (FRAA) status; (**B**) *Borrelia* species infection confirmation among sFR-positive patients (*n* = 8). All 8 sFR-positive patients were VBD-positive (100% concordance). In total, 7 of the 8 patients (87.5%) had confirmed evidence of *Borrelia* species infection (Lyme disease or TBRF). The remaining patient carried an indeterminate TBRF IgG result. sFR = soluble folate receptor, detected when the blocking assay identifies more bound folate than the FRα protein added; VBD = vector-borne disease (IGeneX Laboratories); TBRF = tick-borne relapsing fever; FRAAs = folate receptor alpha autoantibodies; CI = confidence interval (Wilson method).

Of the 68 VBD-positive patients, 8 (11.8%; 95% CI: 6.1–21.5%) were sFR-positive. All 8 sFR-positive patients were FRAA-positive (Figure 1). Among the 8 sFR-positive patients, 7 (87.5%; 95% CI) had confirmed evidence of *Borrelia* species infection, encompassing both Lyme disease and TBRF *Borrelia* species. The remaining patient carried an indeterminate TBRF IgG result.

### 3.4. Infection Profile Among FRAA/VBD+ Patients

Among the 42 FRAA-positive, VBD-positive patients, 24 (57.1%) had evidence of a single vector-borne pathogen, 12 (28.6%) were positive for two pathogens, 4 (9.5%) for three, and 2 (4.8%) for four pathogens concurrently.

**Figure 2:**
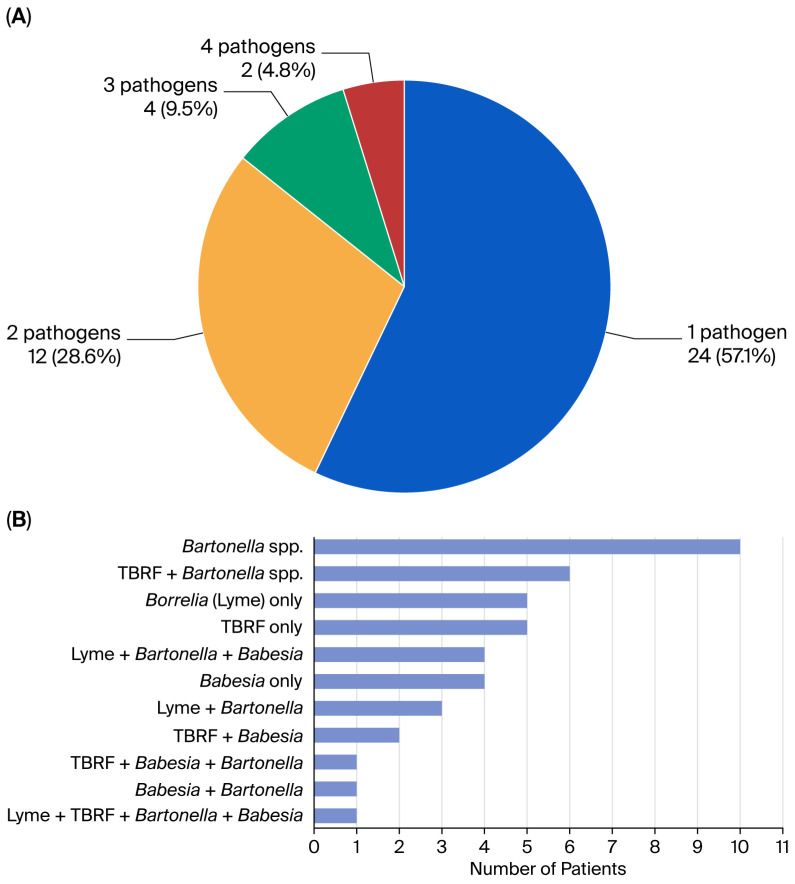
Pathogen distribution and co-infection burden among FRAA+/VBD+ patients evaluated at Lindsey Wells ND, LLC, Wilton and Trumbull, Connecticut, January 2022–August 2025 (*N* = 42). (**A**) Co-infection burden by number of simultaneous pathogens identified; (**B**) specific infection type breakdown by pathogen or pathogen combination. FRAAs = folate receptor alpha autoantibodies; VBD = vector-borne disease (IGeneX Laboratories); TBRF = tick-borne relapsing fever; Lyme refers to *Borrelia burgdorferi* seropositivity. Percentages reflect the proportion of the FRAA+/VBD+ group (*N* = 42).

The most prevalent single pathogen was *Bartonella* spp. alone (*n* = 10, 23.8%), followed by coinfection with *Borrelia* spp. causing TBRF and *Bartonella* spp. (*n* = 6, 14.3%). Lyme disease alone and TBRF alone each accounted for five cases (11.9% each). Among patients with three or more co-infections, the combination of Lyme disease, *Bartonella* spp., and *Babesia* spp. was the most common (*n* = 4; 9.5%), followed by *Babesia* spp. alone (*n* = 4; 9.5%). Lyme disease and *Bartonella* spp. co-infection was identified in three patients (7.1%), and TBRF with *Babesia* spp. in two patients (4.8%). The remaining combinations of TBRF with *Babesia* spp. and *Bartonella* spp., *Babesia* spp. with *Bartonella* spp., and the quadruple co-infection of Lyme disease, TBRF, *Bartonella* spp., and *Babesia* spp., each occurred in a single patient (2.4% each) (Figure 2B).

### 3.5. Association of Neuropsychiatric Symptoms with FRAA Status

**Figure 3:**
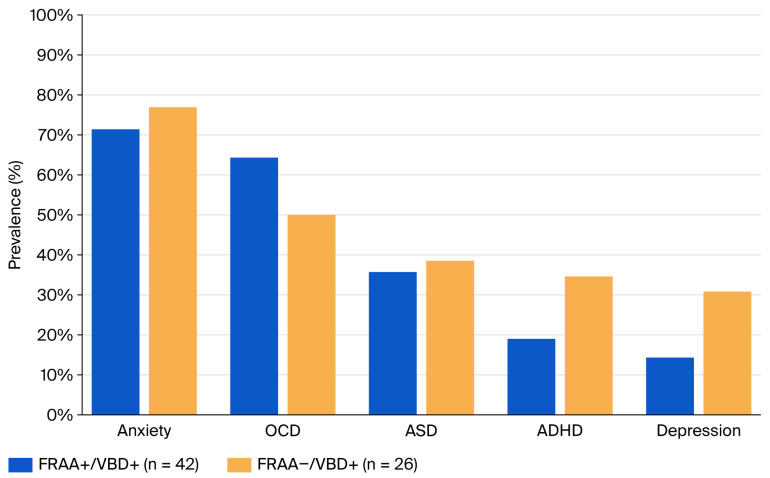
Symptom prevalence by FRAA status (%).

Across the full cohort, anxiety was the most prevalent symptom (50/68; 73.5%), followed by OCD (40/68; 58.8%), ASD (25/68; 36.8%), ADHD (17/68; 25.0%), and depression (14/68; 20.6%) (Figure 3).

Among FRAA-positive patients (*n* = 42), anxiety and OCD symptoms were the most common (71.4% and 64.3%, respectively), followed by ASD (35.7%), ADHD (19.0%), and depression (14.3%). In the FRAA-negative group (*n* = 26), anxiety (76.9%), ADHD (34.6%), and depression (30.8%) were more prevalent compared to the FRAA-positive group. Fisher’s exact tests comparing symptom prevalence between the two groups revealed no statistically significant differences for any domain (all *p* > 0.05; Table 2).

### 3.6. PANS/PANDAS Diagnosis and Association with FRAA Status

Of the 68 patients, 32 (47.1%; 95% CI: 35.7–58.8%) carried a PANS/PANDAS diagnosis. Among the FRAA-positive patients (*n* = 42), 24 (57.1%; 95% CI: 42.2–70.9%) met the criteria for PANS/PANDAS, compared to 8 of the 26 FRAA-negative patients (30.8%; 95% CI: 16.5–50.0%). FRAA-positive patients were significantly more likely to carry a PANS/PANDAS diagnosis than FRAA-negative patients (Fisher’s exact test: *p* = 0.046; OR = 3.00, 95% CI: 1.07–8.43) (Table 3).

## 4. Discussion

This retrospective cohort study is the first to characterize FRAA prevalence in patients with VBDs. Three key findings emerge. First, FRAA prevalence among VBD-positive patients was strikingly high at 61.8% (95% CI: 49.9–72.4%), substantially exceeding published general population rates. Second, the soluble folate receptor (sFR) variant was strongly concentrated in patients with confirmed *Borrelia* species infection (87.5%), suggesting a potential mechanistic link between spirochetal infection and folate receptor autoimmunity. Third, FRAA-positive patients demonstrated a significantly higher rate of PANS/PANDAS diagnoses compared with FRAA-negative patients (57.1% vs. 30.8%; OR = 3.00, 95% CI: 1.07–8.43; *p* = 0.046), identifying a distinct neuropsychiatric subgroup within the VBD population who may benefit from targeted folinic acid supplementation.

The 61.8% FRAA prevalence in patients with VBDs substantially exceeds the 5–15% background rate reported in the general population [7] and approaches the rate associated with ASD cohorts [5,6]. This elevation suggests that FRα autoimmunity may be intertwined with the neuropsychiatric morbidity of VBDs rather than representing a coincidental finding. Given that CFD due to FRAAs is a well-documented and treatable contributor to TRD [11], refractory schizophrenia [13], and ASD [5,6], all conditions in which leucovorin has produced clinically meaningful improvement [12], the high FRAA prevalence observed here identifies a potentially reversible contributor to the neuropsychiatric burden of VBDs.

The 11.8% prevalence of sFR is approximately twice the ~5% rate estimated in general and ASD populations, and its concentration among *Borrelia*-infected patients (7 of 8; 87.5%) is particularly striking. sFRs are circulating folate-binding proteins that do not mediate cellular folate uptake; when present, they sequester folate in circulation and may compound the folate-transport deficit caused by blocking FRAAs. The co-occurrence of sFR with *Borrelia* infection raises the possibility that spirochetal infection induces or amplifies sFR shedding, analogous to the association between sFR and malignancy or inflammation described in the cancer literature [36,37]. Because children with ASD and detectable sFR have been shown to exhibit more severe symptoms and to require higher leucovorin doses to achieve therapeutic benefit [36], patients with VBDs and sFRs may warrant similarly individualized dosing.

The significantly higher prevalence of PANS/PANDAS diagnoses among FRAA-positive patients (57.1% vs. 30.8%; OR = 3.00) is consistent with prior reports linking FRAAs to PANS/PANDAS and extends that association to a VBD-predominant cohort. Neuropsychiatric symptom profiles did not differ significantly between FRAA-positive and FRAA-negative patients (all *p* > 0.05). This absence of a distinguishing phenotype suggests that clinical presentation alone is insufficient to identify affected individuals and supports the case for biochemical screening in patients with VBDs and neuropsychiatric symptoms, rather than reserving testing for specific symptom constellations. Whether FRAA-positive patients experience greater symptom severity or treatment resistance remains an open question that prospective studies with validated severity instruments should address.

Mechanistically, several plausible pathways could link VBDs to FRα autoimmunity. *Borrelia*, *Bartonella*, and *Babesia* spp. all elicit sustained systemic inflammation, and Lyme neuroborreliosis is associated with blood–brain barrier disruption and chronic glial activation [25,26]. Chronic antigenic stimulation together with molecular mimicry between pathogen-derived and folate-receptor epitopes could promote loss of tolerance to FRα, although this remains unproven. The observed concentration of sFR in *Borrelia*-positive patients further suggests a mechanistic role for spirochetal infection, warranting dedicated immunologic and proteomic investigation.

Clinically, these findings argue for incorporating FRAA testing into the evaluation of patients with VBDs, particularly those with neuropsychiatric symptoms that persist despite antimicrobial therapy. Leucovorin crosses the blood–brain barrier through reduced folate carriers independent of FRα, restores cerebrospinal-fluid folate levels in patients with FRAA-positive CFD, and has produced symptomatic improvement in treatment-resistant depression [11], schizophrenia [13], and ASD [5,6]. In the subset of VBD patients with detectable FRAAs, leucovorin could therefore complement antimicrobial treatment and targeted psychiatric care.

This study has several important limitations. The sample size (*n* = 68) limits statistical power, particularly for subgroup analyses. The single-site retrospective design introduces selection bias; patients in this clinical practice were evaluated because of persistent neuropsychiatric symptoms and may not reflect the broader VBD population. The cross-sectional design precludes causal inference: FRAAs and sFRs may be consequences rather than contributors to neuropsychiatric morbidity, and the temporal relationship between VBD onset and FRAA emergence was not established by this study. Symptom assessment relied on retrospective chart review rather than validated severity instruments, limiting evaluation of symptom severity and treatment response. The cohort lacked a non-VBD comparator group; the observed FRAA prevalence is interpreted against published general-population rates, including data from the same clinical practice, which provides a degree of consistency in patient population and diagnostic methodology. VBD positivity was determined using IGeneX Laboratories criteria, which apply IgG thresholds less stringent than CDC two-tier testing guidelines; while this approach reflects standard practice in clinically complex tick-borne disease populations and maximizes sensitivity, it may reduce diagnostic specificity and increase the possibility of false-positive VBD classification. The absence of cerebrospinal fluid 5-methyltetrahydrofolate measurements precludes formal confirmation of CFD; however, serum FRAA testing represents the established and less invasive first-line screening approach, and lumbar puncture carries procedural risks that are disproportionate given that empirical leucovorin treatment is safe and well-tolerated. The association between FRAA positivity and PANS/PANDAS diagnoses (*p* = 0.046) should be interpreted with caution. Diagnoses were made by treating clinicians without structured or blinded assessment, and the borderline *p*-value in the context of a modest sample size warrants careful consideration. Despite these limitations, as the first study to characterize FRAA prevalence in VBDs, this study provides a foundation for larger prospective cohorts, matched case–control studies, and controlled trials of leucovorin augmentation in this population.

## 5. Conclusions and Recommendations

This retrospective cohort study is the first to document a markedly elevated prevalence of FRAAs (61.8%) in patients with VBDs, with FRAA-positive patients carrying three-fold-greater odds of a PANS/PANDAS diagnosis (OR = 3.00; 95% CI: 1.07–8.43; *p* = 0.046). The concentration of sFR positivity among patients with confirmed *Borrelia* species infection (87.5%) further suggests that spirochetal infection may contribute to folate receptor autoimmunity through a distinct mechanistic pathway.

These findings have immediate clinical implications. FRAA-mediated CFD is treatable with leucovorin, which has produced meaningful symptomatic improvement in TRD, schizophrenia, and ASD. The high FRAA prevalence observed here identifies a potentially reversible contributor to the disease burden of VBD that warrants routine consideration in clinical evaluation. FRAA testing should be considered in all VBD patients, especially those who fail to improve with antimicrobial therapy alone.

Larger prospective studies are needed to confirm these prevalence estimates in broader VBD populations, determine whether FRAA-positive patients experience greater neuropsychiatric severity, and clarify the mechanistic relationship between spirochetal infection and folate receptor autoimmunity. Most importantly, given the treatability of CFD, controlled trials of leucovorin augmentation in FRAA-positive VBD patients represent a critical next step in determining whether targeted treatment can meaningfully reduce morbidity in this population.

## Figures and Tables

**Table 1:** Demographic and clinical characteristics of VBD-positive patients with and without folate receptor alpha autoantibodies (FRAAs), evaluated at Lindsey Wells ND, LLC, Wilton and Trumbull, Connecticut, January 2022–August 2025 (*N* = 68).

Characteristic	*n*	%
Sex		
Male	43	63.2%
Female	25	36.8%
Age (years)		
Mean ± SD	12.8 ± 6.7	
Median (range)	13 (2–31)	
Age group		
<6 years	10	14.7%
6–12 years	23	33.8%
13–18 years	21	30.9%
>18 years	14	20.6%
Pediatric (<18 years)	50	73.5%
VBD testing laboratory		
IGeneX Laboratories	68	100%
FRAA testing laboratory		
Religen Laboratories	68	100%
FRAA status		
FRAA-Positive	42	61.8%
FRAA-Negative	26	38.2%
Soluble folate receptor (sFR) status		
sFR-Positive (subset of FRAA+)	8	11.8%
sFR-Negative	60	88.2%

FRAAs = folate receptor alpha autoantibodies. VBD = vector-borne disease. VBD status determined by IGeneX panel testing including serology and fluorescence in situ hybridization (FISH). There was a 95% CI for FRAA+: 49.9–72.4%; for sFR+: 6.1–21.5% (Wilson score method). sFR-positive patients area subset of the FRAA-positive group.

**Table 2:** Neuropsychiatric symptom prevalence by FRAA status (*N* = 68).

Symptom	FRAA+/VBD+ (*n* = 42)	FRAA−/NBD+ (*n* = 26)	Total Cohort (*N* = 68)	*p*-Value *
Anxiety	71.4% (30/42)	76.9% (20/26)	73.5% (50/68)	0.602
OCD	64.3% (27/42)	50.0% (13/26)	58.8% (40/68)	0.467
ASD	35.7% (15/42)	38.5% (10/26)	36.8% (25/68)	0.800
ADHD	19.0% (8/42)	34.6% (9/26)	25.0% (17/68)	0.155
Depression	14.3% (6/42)	30.8% (8/26)	20.6% (14/68)	0.123
Mean symptoms (of 5)	1.87	2.22	2.00	-

* Fisher’s exact test, FRAA+ vs. FRAA−; all comparisons *p* > 0.05. FRAAs = folate receptor alpha autoantibodies. VBD = vector-borne disease. OCD = obsessive–compulsive disorder. ASD = autism spectrum disorder. ADHD = attention-deficit/hyperactivity disorder.

**Table 3:** PANS/PANDAS diagnosis by FRAA status (*N* = 68).

FRAA Status	PANS/PANDAS+	PANS/PANDAS−	Total
FRAA-Positive (*n* = 42)	24 (57.1%)	18 (42.9%)	42
FRAA-Negative (*n* = 26)	8 (30.8%)	18 (69.2%)	26
Total (*N* = 68)	32 (47.1%)	36 (52.9%)	68

Odds Ratio: 3.00 (95% CI: 1.07–8.43); Fisher’s exact test: *p* = 0.046 *; * Statistically significant at *p* < 0.05. PANS = pediatric acute-onset neuropsychiatric syndrome. PANDAS = pediatric autoimmune neuropsychiatric disorders associated with streptococcal infections. FRAAs = folate receptor alpha autoantibodies. Diagnosis ascertained by Lindsey Wells, ND at initial consult or by another provider prior to initial consult. There was a 95% CI for FRAA+ PANS/PANDAS prevalence: 42.2–70.9%; FRAA−PANS/PANDAS prevalence: 16.5–50.0%; overall PANS/PANDAS prevalence: 35.7–58.8%. Fisher’s exact test used for group comparison. Woolf method used for OR calculation.

## Data Availability

The data presented in this study are available on request from the corresponding author. The data are not publicly available due to patient privacy and confidentiality restrictions in accordance with the Health Insurance Portability and Accountability Act (HIPAA).
